# Blood BDNF Level Is Gender Specific in Severe Depression

**DOI:** 10.1371/journal.pone.0127643

**Published:** 2015-05-26

**Authors:** Anatoly Kreinin, Serah Lisson, Elimelech Nesher, Jenny Schneider, Josef Bergman, Kamal Farhat, Joseph Farah, Flavio Lejbkowicz, Gal Yadid, Leon Raskin, Igor Koman, Albert Pinhasov

**Affiliations:** 1 Maale HaCarmel Mental Health Center, Tirat HaCarmel, affiliated to Rappaport Faculty of Medicine, Technion, Israel Institute of Technology, Haifa, Israel; 2 Rappaport Faculty of Medicine, Technion, Israel Institute of Technology, Haifa, Israel; 3 Department of Molecular Biology, Ariel University, Ariel, Israel; 4 Faculty of Life Sciences, Bar-Ilan University, Ramat Gan, Israel; 5 The Nazareth Hospital, EMMS, affiliated with Faculty of Medicine, Bar Ilan University, Nazareth, Israel; 6 Department of Community Medicine and Epidemiology, Carmel Medical Center, Haifa, Israel; 7 Clalit Health Services, National Cancer Control Center, Haifa, Israel; 8 Division of Epidemiology, Department of Medicine, Vanderbilt University, Nashville, Tennessee, United States of America; Peking University, CHINA

## Abstract

Though the role of brain derived neurotrophic factor (BDNF) as a marker for major depressive disorder (MDD) and antidepressant efficacy has been widely studied, the role of BDNF in distinct groups of patients remains unclear. We evaluated the diagnostic value of BDNF as a marker of disease severity measured by HAM-D scores and antidepressants efficacy among MDD patients. Fifty-one patients who met DSM-IV criteria for MDD and were prescribed antidepressants and 38 controls participated in this study. BDNF in serum was measured at baseline, 1st, 2nd and 8th treatment weeks. Depression severity was evaluated using the Hamilton Rating Scale for Depression (HAM-D). BDNF polymorphism rs6265 (val66met) was genotyped. We found a positive correlation between blood BDNF levels and severity of depression only among untreated women with severe MDD (HAM-D>24). Serum BDNF levels were lower in untreated MDD patients compared to control group. Antidepressants increased serum BDNF levels and reduced between-group differences after two weeks of treatment. No correlations were observed between BDNF polymorphism, depression severity, duration of illness, age and BDNF serum levels. Further supporting the role of BDNF in the pathology and treatment of MDD, we suggest that it should not be used as a universal biomarker for diagnosis of MDD in the general population. However, it has diagnostic value for the assessment of disease progression and treatment efficacy in individual patients.

## Introduction

Major depressive disorder (MDD) is a chronic and debilitating syndrome [1,2], ranked as a global disease burden [3]. MDD is described in the DSM-IV as a complex and heterogeneous disorder characterized by a wide spectrum of symptoms that often complicates diagnosis and treatment decision making [4]. The etiology of MDD is not completely understood, but evidence supports the involvement of genetic and environmental factors [5–7]. In the absence of valid biomarkers for diagnostic and treatment efficacy, a number of subjective clinician- and patient-rated instruments have been developed, including the clinician-administered Hamilton Rating Scale for Depression (HAM-D) [8]. According to Handbook of Clinical Rating Scales and Assessment in Psychiatry and Mental Health [9], MDD patients can be divided by HAM-D scores into the following groups: from 0 to 6—no depression, from 7 to 17—mild depression, from 18 to 24—moderate depression, and scores over 24 indicate severe depression. The group of severe MDD patients requires special attention because of high resistance to the existing therapy and, increased suicide attempts and mortality [10]. Numerous studies have attempted to identify biochemical and molecular markers that might help in the diagnosis of depression and in monitoring efficacy of antidepressant treatment [11–13]. One of the most studied marker candidates is a gene encoding Brain Derived Neurotrophic Factor (BDNF)—a neurotrophin presented in both central and peripheral nervous systems [14–18]. BDNF is known for its role in the regulation of neuronal differentiation, outgrowth, repair and survival as well as synaptic connectivity [19–23]. Accumulating evidence has shown blood BDNF levels to be lower in MDD patients compared to control subjects [24–28]. Some studies show that blood BDNF levels in untreated MDD patients associate with MDD severity [29,30], while others do not support this claim [25,27,31] which leaves a gap for further investigation of the relationship between BDNF blood levels and the severity of MDD [25,32]. There is general agreement that antidepressants modulate BDNF levels via stimulation of cAMP response element binding protein (CREB) function [33,34]. In patients treated with different antidepressants increased peripheral blood BDNF levels were observed [35,36]. Because BDNF can pass the blood brain barrier [37], its blood levels can be used for the assessment of its levels in the brain.

Among factors contributing to its variability are single nucleotide polymorphisms (SNPs), including the widely studied val66met (rs6265). Some studies have reported an association between BDNF polymorphisms and symptoms of depression [38–40], while others have shown no association [41–45]. Numerous studies explored the association between the BDNF polymorphism and/or blood BDNF levels in terms of ethnical groups [46–49], age [39,50] and gender [27,46,50–53] further investigations are warranted [27,52,53]. Particularly, it was shown that among Caucasians, the frequency of the Met allele is 25–32%, while among Asians the Met allele is more frequent, reaching 40–50% [48,49], however meta-analysis studies did not support the link between BDNF val/met polymorphism and risk of MDD [46]. While, two large population-based studies, have demonstrated gender difference in the lifetime prevalence of MDD of about 21% in women and around 11% in men [54], a link between gender and BDNF polymorphism and its blood levels has not been fully elucidated and requires further evaluation.

The goal of this study was to evaluate the diagnostic value of BDNF as a potential gender-dependent marker of MDD severity and antidepressant treatment efficacy. To achieve this goal we examined the association between BDNF serum levels, BDNF polymorphism, and severity of clinical symptoms in a cohort of MDD patients before and during different stages of treatment with antidepressants.

## Subjects and Methods

### Subjects

Eighty-nine volunteers (51 MDD patients and 38 healthy controls) participated in an 8-week, case-control, 2-center study. Fifty-one patients (17 men, 34 women) aged 25 to 64 years (44.75 ± 11.47) that met Diagnostic and Statistical Manual of Mental Disorders, Fourth Edition, Text Revision (DSM-IV-TR) criteria for MDD were recruited from the outpatient and inpatient clinics of Maale HaCarmel Mental Health Center and the Nazareth Hospital-EMMS in Israel. All patients were physically healthy and had no unstable medical conditions that would require treatment and adjustment of dosages of prescribed medications, no significant medical or neurological illnesses, pregnancy or treatment with any steroid or hormonal supplement. In addition, none had comorbidity with other psychiatric disorders, as confirmed by DSM-IV and none had taken any psychotropic medication within a month prior to initiation of the study. The study included 38 healthy controls (16 men, 22 women) aged 21 to 58 years (34.71±10.56). Inclusion criteria for the control group were good physical health, no history of mental disorders and no treatment with any psychotropic agents during the previous 3 months.

The study was approved by Maale HaCarmel Mental Health Center, Nazareth Hospital-EMMS and the National Ministry of Health Ethical Review Boards. The patients enrolled to the study after they received a comprehensive explanation of study procedures and provided written informed consent. The study was recorded in http://clinicaltrials.gov/show/NCT00944996 (study number NCT00944996).

### The evaluation of depression severity

At the initial screening visit, a thorough clinical and psychiatric examination was performed on patients who met entry criteria. The HAM-D was used to measure symptoms and severity of the disorder at baseline and after 1, 2 and 8 weeks of treatment. Senior psychiatrists (one physician at each site) enrolled and established patients’ diagnoses according to DSM-IV criteria. All participants began treatment with antidepressants at the initial screening visit. Prior to the study, raters were trained and reached acceptable levels of inter-rater reliability, estimated by intraclass correlation coefficient (ICC), for the primary diagnosis, HAM-D (ICC = 0.86).

### Medication

Patients were prescribed medication by their psychiatrists according to disease severity as measured by HAM-D (18 and higher) that was used as a cut off point for the both inclusion in the study and initiation of treatment. Patients were treated with the following selective serotonin reuptake inhibitors (SSRIs): 20 with fluoxetine (20 mg/day), 13 with escitalopram (10 mg/day) and 9 with paroxetine (20 mg/day). Nine patients were treated with the serotonin-norepinephrine reuptake inhibitor (SNRI) venlafaxine (up to 225 mg/day).

### BDNF measurement

The evaluation of serum BDNF was blind and performed in both patient and control groups at four time points: baseline and after week 1, week 2, and week 8. The purpose of the BDNF measurement in controls at four time points was to assess time-dependent changes in the control group. A blood sample (5 ml) was obtained from the antecubital vein between 8 am and 10 am and was collected into anticoagulant-free tubes. Blood samples were kept at room temperature for 1 hour, followed by 1 hour at 4°C, and then centrifuged at 3500g x 15 minutes at 4°C. Serum was collected and kept at -80°C before analysis. The BDNF assay was performed using a solid-phase, sandwich, two-site, enzyme-linked immunoassay (ELISA) (Promega, USA Cat#G7610), according to the manufacturer’s instructions. All samples were assayed in duplicate and the mean was calculated.

### Genotyping of BDNF variant

Genomic DNA was extracted using a commercially available kit according to the manufacturer's protocol (Puregene DNA extraction kit; Gentra Systems). Genotyping of BDNF rs6265 (val66met) was performed using Restriction Fragment Length Polymorphism (RFLP) assay.

### Statistical analysis

The statistical significance between groups was assessed using *t*-test, two tailed Pearson correlation analysis, and multivariate linear regression model implemented in SAS 9.4 and GraphPad Prism 5.02.

## Results

### Characteristics of the study participants

The demographics of study participants are presented in Table 1. Age of the participants was significantly lower among controls than among MDD patients (*p*<0.001). There were no other significant differences between MDD patients and the control group. Over 84% of patients (n = 43) had severe MDD defined by HAM-D-score>24. Dropout rate for the treatment group was as following: 9 patients at second appointment (one week of treatment), 2 patients at third appointment (two weeks of treatment), and 5 patients at fourth appointment (eight weeks of treatment).

**Table 1:** Demographics of the 89 study participants.

	Patients (51)	Control (38)	*p*-value
**Age (years ± SD)**	44.75 ± 11.47	34.71±10.56	<0.001
**Gender**			
male	17	16	0.532
female	34	22	
**Duration of disease (median in years)**	5	--	--
**Number of episodes (median)**	2	--	--
**HAM-D scores**			
moderate (18–24)	7	--	--
severe (>24)	44	--	
**Ethnicity/ ethnic group**			
Jews	41	35	0.213
Arabs	10	3	
**Marital status**			
Married	35	22	
Divorced	8	5	0.993
Single	5	11	
Widowed	3	0	

### Correlation between HAM-D and serum BDNF levels at baseline

**Fig 1:**
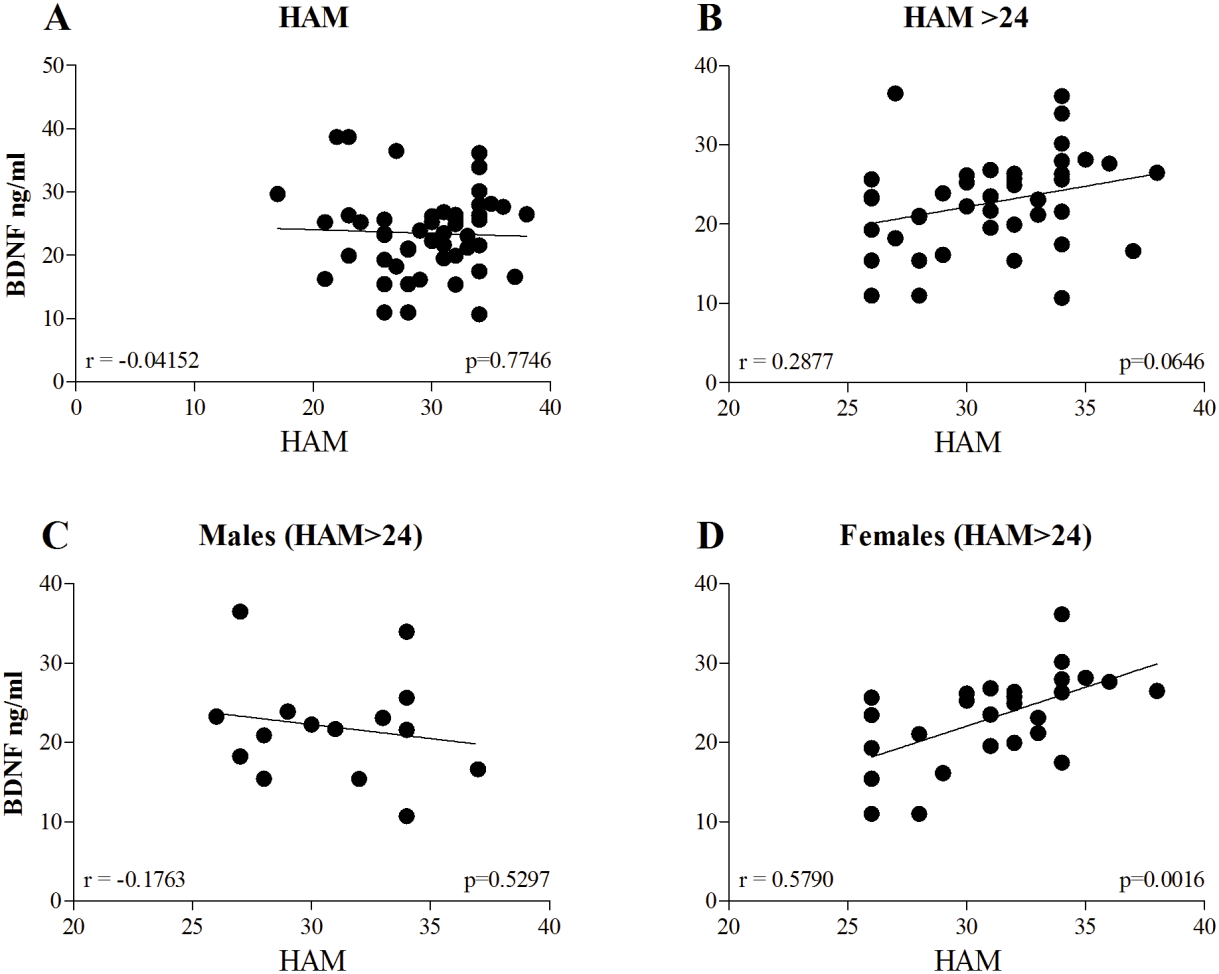
Scatter plot of the correlation between serum BDNF levels and HAM-D scores at baseline. Data represent entire results (A), results obtained with HAM-D>24 excluding criteria (see Results section) applied to all patients (B) as well as to males (C) and females (D) separately. Statistical significance between groups for each time point was assessed using two tailed Pearson correlation analysis, indicated by *p*-value.

We analyzed the relationship between baseline BDNF levels and HAM-D scores and found no correlation within the entire range of HAM-D scores (Fig 1A; *p* = 0.7746, n = 51). Analysis of severe depression cases (HAM-D>24) showed a trend for correlation between baseline BDNF levels and HAM-D scores (Fig 1B; *p* = 0.0646, n = 43). Gender-specific analysis of the cases with severe depression (HAM-D>24) revealed a significant correlation between HAM-D and BDNF serum levels at the entry point among women (Fig 1D; *p* = 0.0016 *p*<0.01, n = 28), but no correlation was observed in men (Fig 1C; *p* = 0.5297, n = 15). We also performed a multivariate linear regression analysis; the model included BDNF levels at baseline, number of episodes, age at diagnosis, duration of disease, ethnicity (Arab/Jewish), and age. The analysis showed statistically significant association between baseline BDNF levels with HAM-D score among women with severe depression (*p* = 0.032), but no association was observed in men (*p* = 0.898). Other variables, including ethnicity, were not significantly associated with HAM-D scores in both men and women.

### BDNF serum levels at entry point and during treatment course

**Fig 2:**
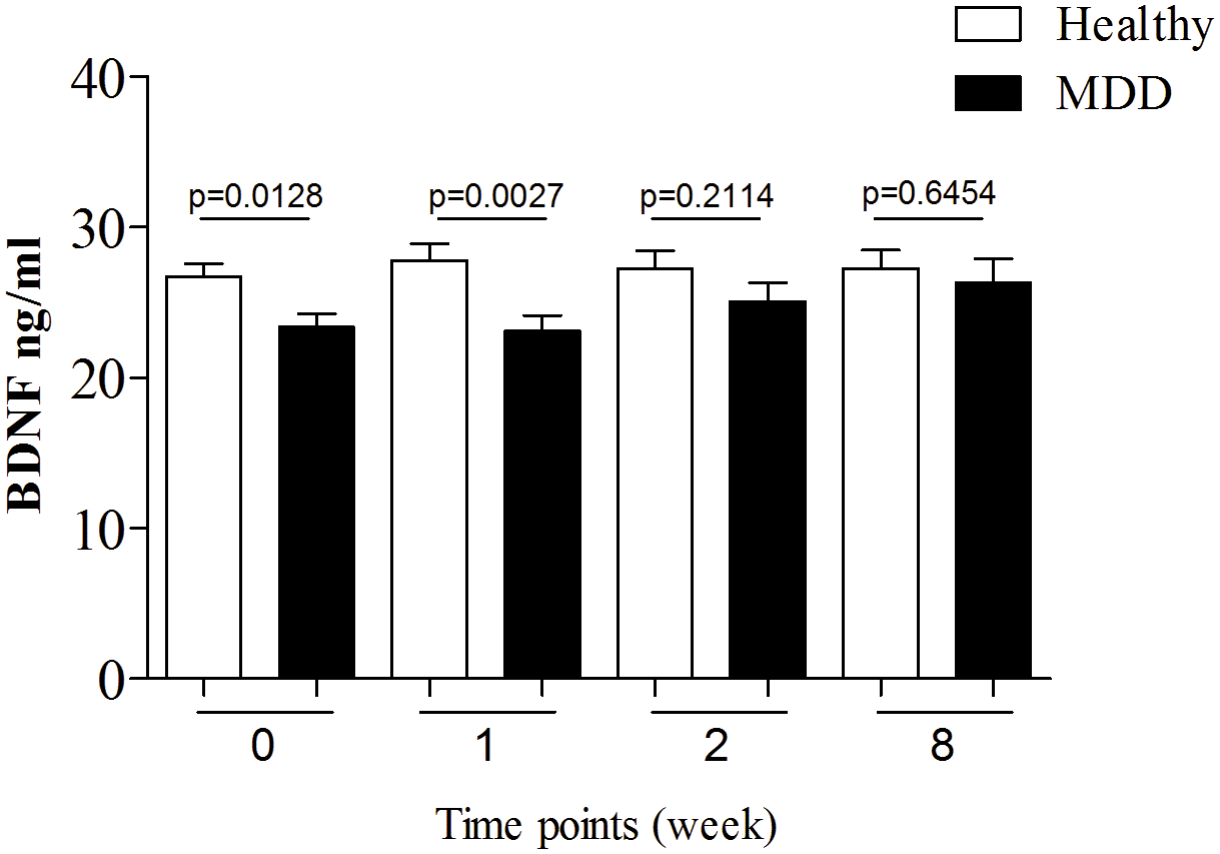
Serum BDNF levels of MDD patients and healthy controls. Data represent four different time points: baseline (0) and 1^st^, 2^nd^ and 8^th^ weeks of treatment respectively. Statistical significance between groups for each time point was assessed using *t*-test, indicated by *p*-value.

A significant difference in serum BDNF levels was observed between MDD patients and healthy controls at entry point (Fig 2; week 0, *p*<0.05). The difference between groups slightly increased one week after the treatment initiation (*p*<0.01) and disappeared after the second week (Fig 2). The MDD group showed a tendency toward increased BDNF levels after the treatment started; no such tendency was observed in the control group. No significant correlations were found between serum BDNF levels and age of onset of MDD, its duration, and number of depressive episodes. In addition, no significant differences were detected between BDNF serum levels and type of antidepressants used in this study.

### BDNF genotyping

Val66met genotypes were equally distributed between MDD cases and controls (Table 2). No association was found between BDNF levels and the genotypes under the additive model of inheritance (*p* = 0.894).

**Table 2:** Distribution of three BDNF genotypes among tested groups (additive model).

val66met	Control, n (%)	MDD, n (%)	Total, n (%)	Odds ratio	*p*-value
val66val	22 (57.89)	31 (60.78)	53 (59.55)		
val66met	15 (39.47)	18 (35.29)	33 (37.08)	0.95	0.864
met66met	1 (2.63)	2 (3.92)	3 (3.37)		

## Discussion

The major finding of this work is a significant positive correlation between blood BDNF levels and depression severity in untreated female patients with severe MDD determined by the HAM-D>24.

In the present study, the initial MDD group included previously untreated patients with HAM-D scores ranging from 17 to 40. Correlation analysis between depression severity and BDNF levels in this group did not reveal any significance (Fig 1A). Stratified analysis of the patients with moderate (HAM-D<24) and severe (HAM-D≥24) MDD showed no significant correlation (*p* = 0.775) between BDNF serum levels and severity of disease in moderate MDD, while patients with severe MDD had a tendency (*p* = 0.064) towards a positive correlation between baseline BDNF levels and depression severity (Fig 1B). Considering known gender differences in depression [51], we analyzed BDNF/HAM-D correlation in untreated men and women with severe MDD (HAM-D≥24) separately. A significant positive correlation between baseline BDNF and disease severity was detected in women (Fig 1D; *p* = 0.0016), but not in men (Fig 1C; *p* = 0.5297). The correlation between BDNF blood levels and depression severity in females was markedly stronger than in the mixed group (Fig 1B).

Findings about the association between BDNF levels and severity of depression are not ubiquitous [31]. The correlation between depression severity and BDNF levels in blood was previously studied on the whole spectrum of MDD patients with respect to HAM-D scores. These studies showed either a negative relationship [24,29,55,56] or no correlation between these two parameters [57]. We believe that the modifying effect of gender and depression severity (evaluated by the HAM-D scale) can explain a controversy between previous studies of the relationship between BDNF levels and disease severity in untreated MDD patients [24,29,55–57]. Our conclusion is partially supported by the fact that depression is more common among women than men [58–60].

We also measured baseline BDNF levels in untreated MDD patients and the control group (Fig 2). We found that BDNF levels in untreated MDD patients are lower than in healthy individuals similar to previous studies [17,24,55,57,61]. Though, several other reports did not observe this phenomenon [62,63], it is important to stress that age difference between MDD patients (44.75 ± 11.47) and controls (34.71±10.56) may impact the BDNF levels at study baseline. However, increase in the blood levels of BDNF after treatment with antidepressants cannot be explained by age difference.

In agreement with previous studies [17,18,24,57], we also found that antidepressants (SSRI and SNRI) raised BDNF levels after the second week of treatment. There were no significant statistical differences in BDNF levels between patients treated with different SSRIs or SNRIs, which concur with a recent report demonstrating an increase in BDNF after SSRI or SNRI treatment [64].

We did not find significant association between val66met and BDNF levels. However, we believe that other genetic variants in BDNF should be considered, especially rare variants with prominent functional impact. Some of the variability between previous studies showing conflicting data on val66met may be explained by ethnicity-specific variants. However, the small sample size of our study may not provide sufficient statistical power to identify the association between BDNF variants and BDNF levels.

Our study has a number of limitations, and generalization of our findings should be done with caution. The main limitation was sample size, which is relatively small and may affect extrapolation of our conclusion. Although the relatively high dropout rate did not affect analysis of BDNF baseline levels as well as genetic parameters, it might affect assessment of the correlation between treatment effect and the dynamics of BDNF levels. Further studies of the gender effect on the correlation between BDNF and depression severity are warranted.

## Conclusion

The present study further supports the role of BDNF in the pathology and treatment of MDD. The most important finding of this study is a positive correlation between serum BDNF levels and depression severity in women with severe MDD (HAM-D≥24). We conclude that BDNF should not be used as a biomarker for screening of MDD in general population. On the other hand, it has certain diagnostic value for the assessment of disorder severity and treatment efficacy in individual patients.
